# Constraints to virus infection in *Nicotiana benthamiana *plants transformed with a potyvirus amplicon

**DOI:** 10.1186/1471-2229-10-139

**Published:** 2010-07-06

**Authors:** María Calvo, Gabriela Dujovny, Cristina Lucini, Jesús Ortuño, Josefa M Alamillo, Carmen Simón-Mateo, Juan José López-Moya, Juan Antonio García

**Affiliations:** 1 Centro Nacional de Biotecnología-CSIC, Campus de la Universidad Autónoma de Madrid, 28049 Madrid, Spain; 2 Facultad de Ciencias y Artes, Universidad Católica de Ávila, Ávila, Spain; 3 Departamento de Fisiología Vegetal, Facultad de Ciencias, Universidad de Córdoba, Córdoba, Spain; 4 Centre for Research in Agricultural Genomics CRAG, CSIC-IRTA-UAB, Barcelona, Spain

## Abstract

**Background:**

Plant genomes have been transformed with full-length cDNA copies of viral genomes, giving rise to what has been called 'amplicon' systems, trying to combine the genetic stability of transgenic plants with the elevated replication rate of plant viruses. However, amplicons' performance has been very variable regardless of the virus on which they are based. This has boosted further interest in understanding the underlying mechanisms that cause this behavior differences, and in developing strategies to control amplicon expression.

**Results:**

*Nicotiana benthamiana *plants were transformed with an amplicon consisting of a full-length cDNA of the potyvirus *Plum pox virus *(PPV) genome modified to include a GFP reporter gene. Amplicon expression exhibited a great variability among different transgenic lines and even among different plants of the same line. Plants of the line 10.6 initially developed without signs of amplicon expression, but at different times some of them started to display sporadic infection foci in leaves approaching maturity. The infection progressed systemically, but at later times the infected plants recovered and returned to an amplicon-inactive state. The failure to detect virus-specific siRNAs in 10.6 plants before amplicon induction and after recovery suggested that a strong amplicon-specific RNA silencing is not established in these plants. However, the coexpression of extra viral silencing suppressors caused some amplicon activation, suggesting that a low level of RNA silencing could be contributing to maintain amplicon repression in the 10.6 plants. The resistance mechanisms that prevent amplicon-derived virus infection were also active against exogenous PPV introduced by mechanical inoculation or grafting, but did not affect other viruses. Amplicon-derived PPV was able to spread into wild type scions grafted in 10.6 rootstocks that did not display signs of amplicon expression, suggesting that resistance has little effect on virus movement.

**Conclusions:**

Our results suggest that amplicon-derived virus infection is limited in this particular transgenic line by a combination of factors, including the presumed low efficiency of the conversion from the transgene transcript to replicable viral RNA, and also by the activation of RNA silencing and other defensive responses of the plant, which are not completely neutralized by viral suppressors.

## Background

The increasing interest in the use of plants as biofactories for the production of foreign proteins of pharmaceutical or industrial importance has led to the development of novel expression systems to obtain high yields of the desired products [1]. Some strategies have been focused on the development of plant viral vectors, taking into consideration their replication capacity as a positive feature for achieving the expected yields [2-4].

Despite the fact that its speediness can sometimes make transient expression to be a convenient method for obtaining the product of interest, one of the aims of molecular farming is to develop stable systems to avoid manual inoculation processes that might hinder large-scale production and increase costs. With this purpose, plant genomes have been transformed with full-length cDNA copies of the genomes of viral vectors, giving rise to what has been called 'amplicon' expression systems [5-11]. Therefore, amplicons, which are virus-derived transgenes, were designed to combine the genetic stability of transgenic plants with the elevated replication rate of plant viruses, escaping the need of an inoculation process. However, amplicons' performance in transgenic lines obtained by transformation of different viral genomes, as a whole, has been very variable regardless of the virus on which they are based. This has boosted further interest in understanding the underlying mechanisms that cause these behavior differences, and in developing strategies to control amplicon expression.

RNA silencing is used by plants as a defense mechanism against virus infections [12], and it also contributes to constrain the replication of viral amplicons [5,13-16]. However, RNA silencing may not be the only factor limiting amplicon expression in many occasions, for instance it has been reported that a mutant version of *Cucumber mosaic virus *(CMV) lacking its silencing suppressor 2b was nevertheless able to alleviate the repression of a *Potato leafroll virus *(PLRV) amplicon [17]. Thus, amplicons can be used not only for molecular farming purposes, but they can also serve as model systems for analyzing transgene expression and virus infection processes, as well as antiviral defense mechanisms.

*Plum pox virus *(PPV) is a member of the genus *Potyvirus *[18]. Potyviruses have a single-stranded RNA genome of messenger polarity ~10 kb in length. This genomic RNA is translated into a large polyprotein and a truncated frameshift product, which are proteolytically processed by three virus-encoded proteinases [19,20]. Full-length cDNA clones that are able to initiate PPV infections have been constructed and used for the genetic characterization of this virus and to design efficient plant expression vectors [21].

Coexpression of the potyviral silencing suppressor HCPro has been shown to strongly enhance the activity of a *Potato virus X *amplicon [16], however it is not known whether its own HCPro expression could secure high levels of expression of a potyviral amplicon. In this work we have transformed *Nicotiana benthamiana *plants with a full-length cDNA copy of the potyvirus PPV. *N. benthamiana*, an experimental host of PPV, was chosen due to its peculiarities that make it a model species in virological studies in plants [22]. We have observed a large variability in the expression patterns of the resulting transgenic lines, and we have characterized in detail the amplicon expression of one of these lines.

## Results

### Production of PPV-NK-GFP amplicon lines

**Figure 1:**
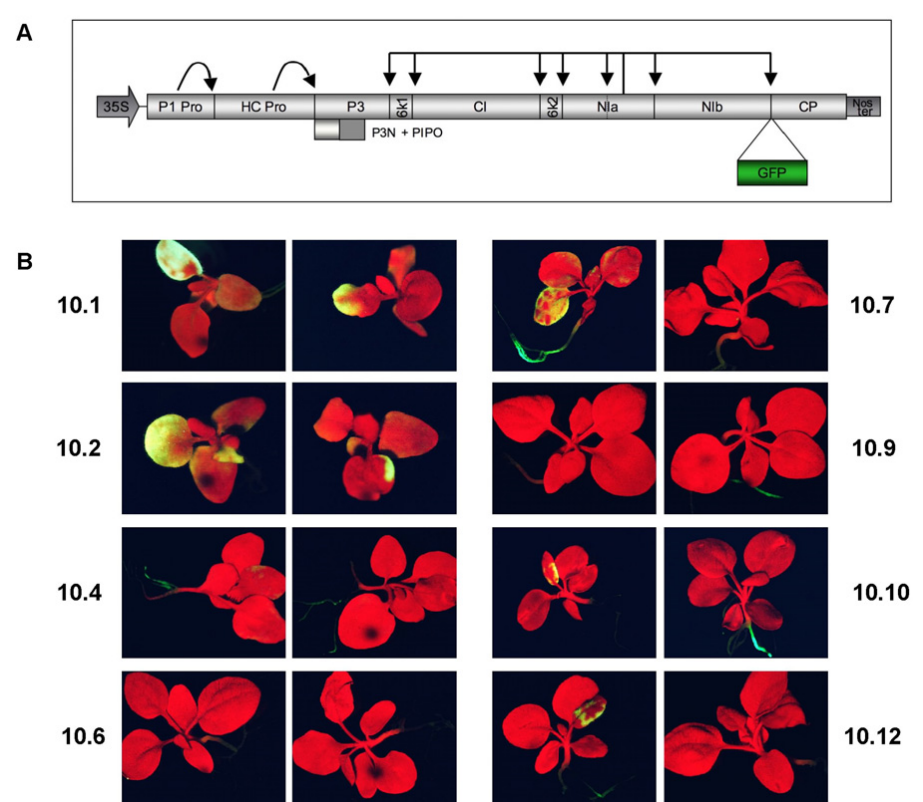
**PPV-NK-GFP amplicon plants**. **A: **Schematic representation of p35S-PPV-NK-GFP-NOSt. Grey boxes represent the coding sequences of the indicated PPV proteins. The green box represent the GFP reporter gene cloned between the NIb and CP coding sequences. Black arrows indicate proteinase cleavage sites. 35S: *Cauliflower mosaic virus *35S promoter. NOSt: terminator of the nopaline synthase gene. **B: **Seedlings of the F1 generation of the different PPV-NK-GFP transgenic lines monitored for GFP expression under UV light.

*N. benthamiana *plants were transformed with the complete cDNA sequence of the potyvirus PPV carrying the coding sequence of the green fluorescent protein (GFP) as a reporter gene (PPV-NK-GFP, Fig. 1).

Plant development, viral accumulation and GFP expression were greatly variable among the primary transformants. In particular, two plants were sterile, and another one (10.2) yielded very few seeds, most of which were not viable. Seeds of fertile transformants were germinated *in vitro *and the resulting seedlings were analyzed for GFP expression. Interestingly, GFP was detected in the only two seedlings of the line 10.2 that could be obtained, and in all the seedlings of the line 10.1, but in the rest of the other fertile lines, GFP accumulation was only observed in some of the seedlings. However, although theoretically all cells of the transformed plants contain the viral transgene, even in seedlings of the line 10.1 the observed GFP expression was not uniform in cotyledons and leaves (Fig. 1B). Data of kanamycin resistance segregation of the different transgenic lines were compatible with either one or two insertion loci, but we did not find correlation between the loci numbers and the GFP expression patterns (data not shown). Seedlings from some of the lines were transferred to soil and cultured in a growth chamber. Patterns of GFP expression were very variable, not only between lines, but also between plants within the same line (data not shown).

### Expression of the PPV-NK-GFP amplicon in the 10.1 and 10.6 lines

**Figure 2:**
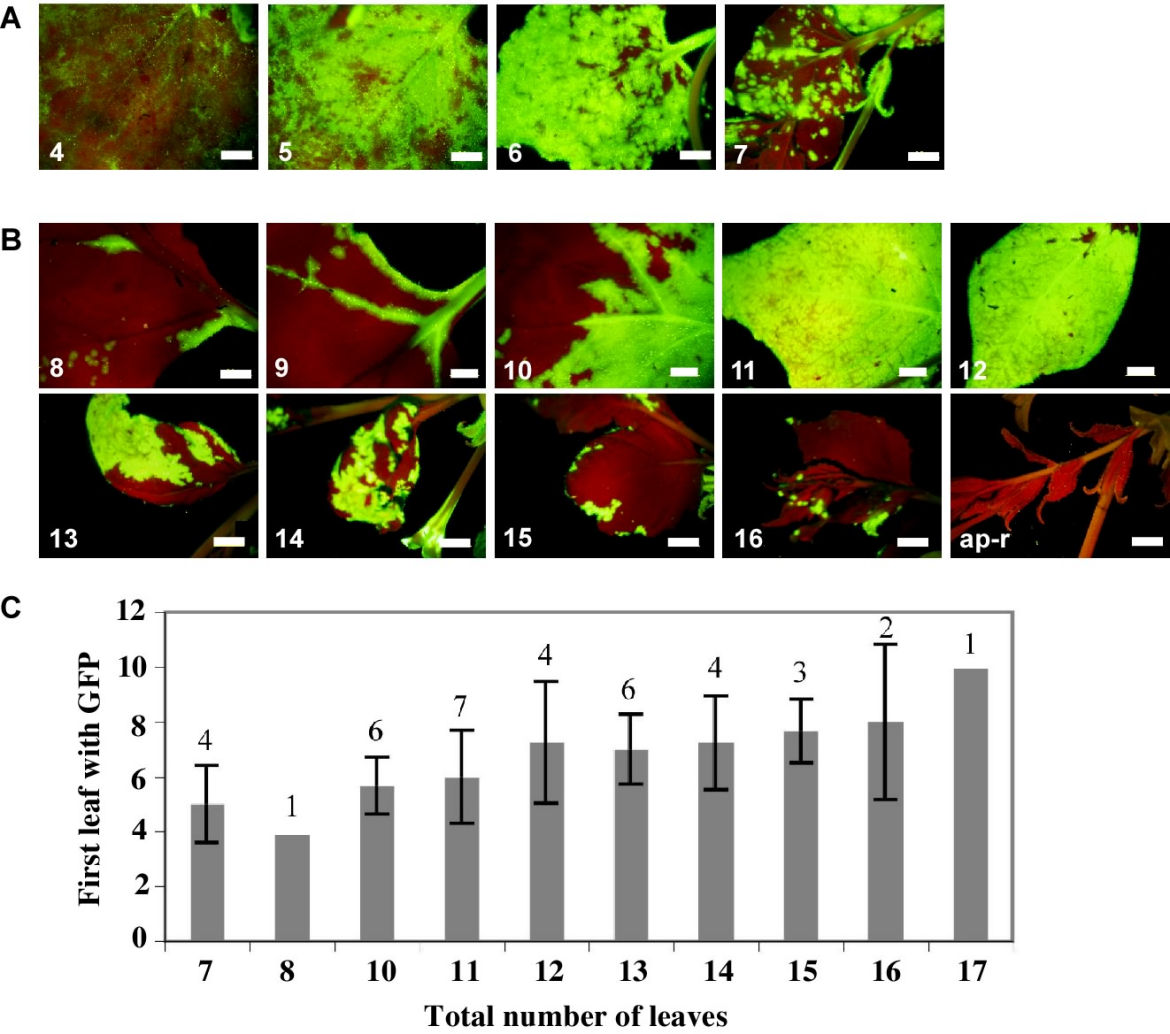
**Expression patterns of the PPV-NK-GFP amplicon in transgenic 10.1.7 (A) and 10.6.1 (B) plants**. Leaf positions (from the bottom of the plant) are indicated. Pictures were taken under UV light at 42 dpt (10.1.7, and leaves 8-10 of 10.6.1) and 50 dpt (leaves 11-16 of 10.6.1). Scale bar: 0.5 cm. Panel **C **summarizes data about the initiation of amplicon expression in 10.6.1 plants. Plants were classified in different groups according to their number of leaves at the time amplicon expression was detected. Bars represent the average and standard deviation of the positions of the first GFP-expressing leaves. The number of plants of each group is shown on top of the bars.

10.1 was the transgenic line that showed the most efficient amplicon expression. Plants from this line accumulated high levels of virus since they sprout, although, even in this case, GFP expression was not evenly distributed on the cotyledons of each seedling, and high variability was also observed from seedling to seedling (Fig. 1B). However, as they grew, all the 10.1 plants displayed strong infection symptoms that caused severe development impairment, and persisted for the complete plant life (Fig. 2A). This infection pattern is similar to that of a normal infection caused by PPV-NK-GFP in wild type *N. benthamiana *plants.

In contrast with line 10.1, none of the seedlings of the line 10.6 expressed GFP initially. However, when these seedlings were transferred to soil, a number of plants started to express GFP after some days in culture. Successful amplicon expression and subsequent viral infection appeared to be a stochastic event in the line 10.6, and this phenotype was maintained in different F2 progenies of this line. The offspring of plant 10.6.1 (F2) was used for the next experiments.

Although amplicon induction took place in different plants at different days post transplantation (dpt), GFP expression always started in expanding leaves undergoing maturity (Fig. 2C shows the results of 5 independent experiments). In the first amplicon-expressing leaves, green fluorescence was detected in isolated foci, which did not progress extensively. GFP expression usually spread to cover the base of leaves just on top of the first GFP-expressing leaf, whereas GFP was detected throughout the complete lamina of younger leaves (Fig. 2B). However, approximately 10 days after the appearance of the first green fluorescent spots, dark green islands typical of virus recovery [23] were observed in newly developing leaves. The induction of the recovery phenotype was confirmed by further emergence of leaves that did not show infection symptoms or GFP expression.

Because of the abnormal expression pattern observed in line 10.6, we focused further study on this specific line.

### Accumulation of virus-specific siRNAs in the PPV-NK-GFP amplicon line 10.6

RNA silencing has been shown to mediate constitutive and virus-induced virus resistance derived from viral transgenes [24-26], and to repress the expression of viral amplicons [5]. In order to assess the possible contribution of RNA silencing to the initial repression of amplicon expression, and to the eventual induction of virus recovery in the 10.6 plants, we analyzed the accumulation of siRNAs specific of the viral transgene, the main hallmark of RNA silencing processes.

**Figure 3:**
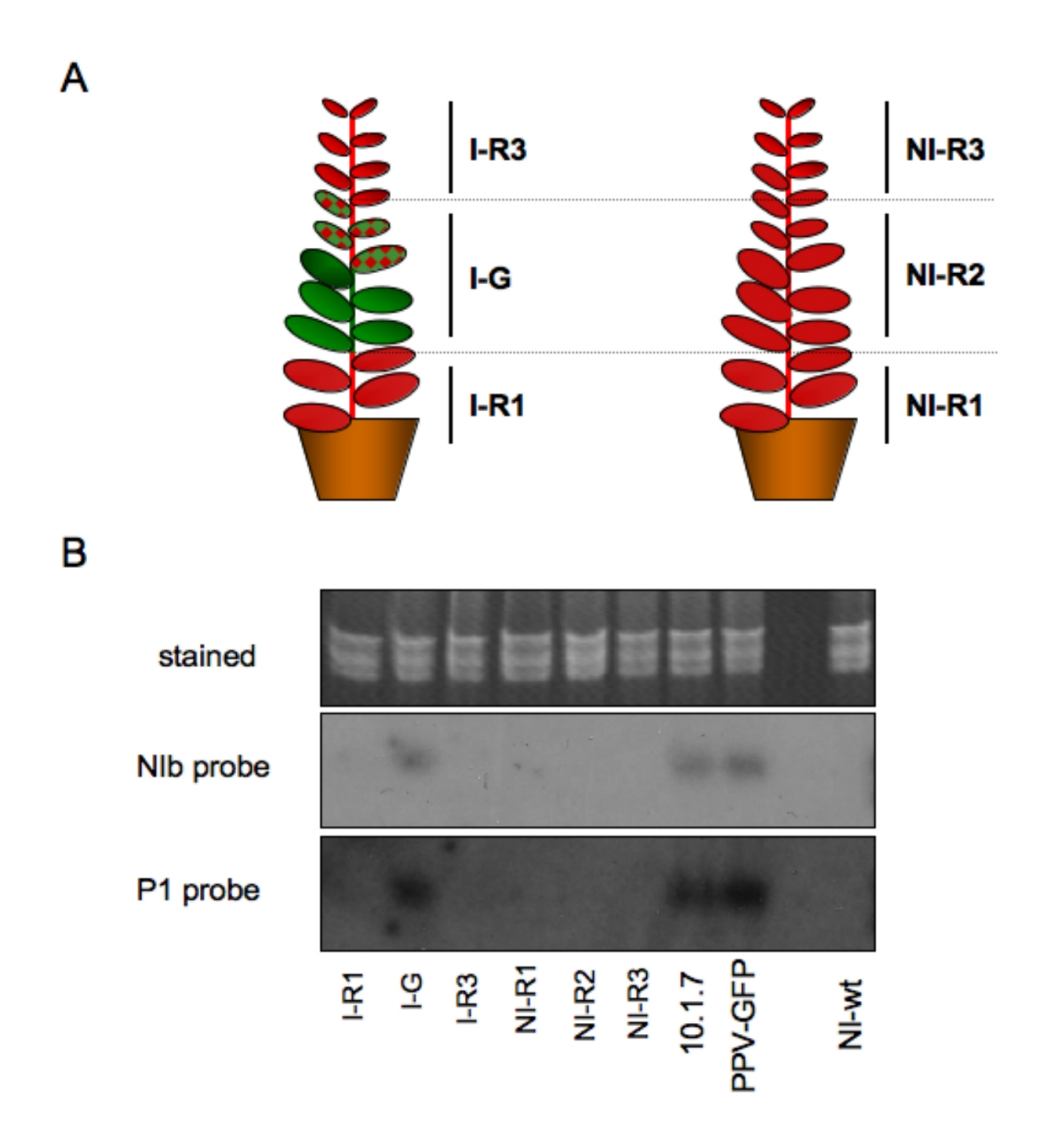
**siRNA accumulation in 10.6.1 amplicon plants**. **A: **Schematic representation of the two kind of 10.6.1 plants. Green and red patterns indicate expression and lack of expression of GFP, respectively. **B**: RNA was extracted from the leaf samples indicated in panel **A**, and subjected to Northern analysis with probes corresponding to the PPV NIb and P1 coding sequences. RNA samples from a 10.1.7 transgenic plant, expressing the PPV-NK-GFP amplicon from germination, and from wild type *N. benthamiana *plants manually infected with PPV-NK-GFP (PPV-GFP) or non-infected (NI-wt) were used as controls. The upper panel shows tRNAs and 5S RNAs stained with ethidium bromide.

Seeds of 10.6.1 plants were germinated *in vitro *and the resulting seedlings were transplanted and cultured in soil. In this particular experiment, 13 out of 64 plants developed an amplicon-derived infection. Small RNAs were extracted from three types of leaves of the infected plants: leaves below the first leaf that expressed GFP (R1); leaves undergoing PPV-NK-GFP infection (G); and fully recovered young leaves (R3). Equivalent samples, R1, R2 (of the same age as G leaves) and R3, were collected from 10.6.1 plants not showing symptoms of infection, and which were maintained in identical growth conditions (Fig. 3A). The small RNA samples were subjected to Northern analysis using probes derived from either the P1 or the NIb coding sequence of PPV (Fig. 3B). PPV-specific siRNAs were only detected in samples from GFP-expressing leaves (G). Viral siRNAs were neither detected in leaves developed before the amplicon induction (R1) nor in upper recovered leaves (R3) of the infected 10.6.1 plants. Viral siRNAs were also not detected in any of the samples collected from the non-infected control plants.

### Effect of RNA silencing suppressors on the expression of the PPV-NK-GFP amplicon 10.6

The absence of detectable siRNAs in the tissue of the 10.6.1 plants that were not expressing the PPV amplicon suggested that RNA silencing was not active in these plants. To confirm that RNA silencing was not playing a role in the repression of the amplicon, 10.6.1 plants that did not display signs of PPV-NK-GFP infection were infiltrated with cultures of *Agrobacterium tumefaciens *expressing different viral silencing suppressors.

**Figure 4:**
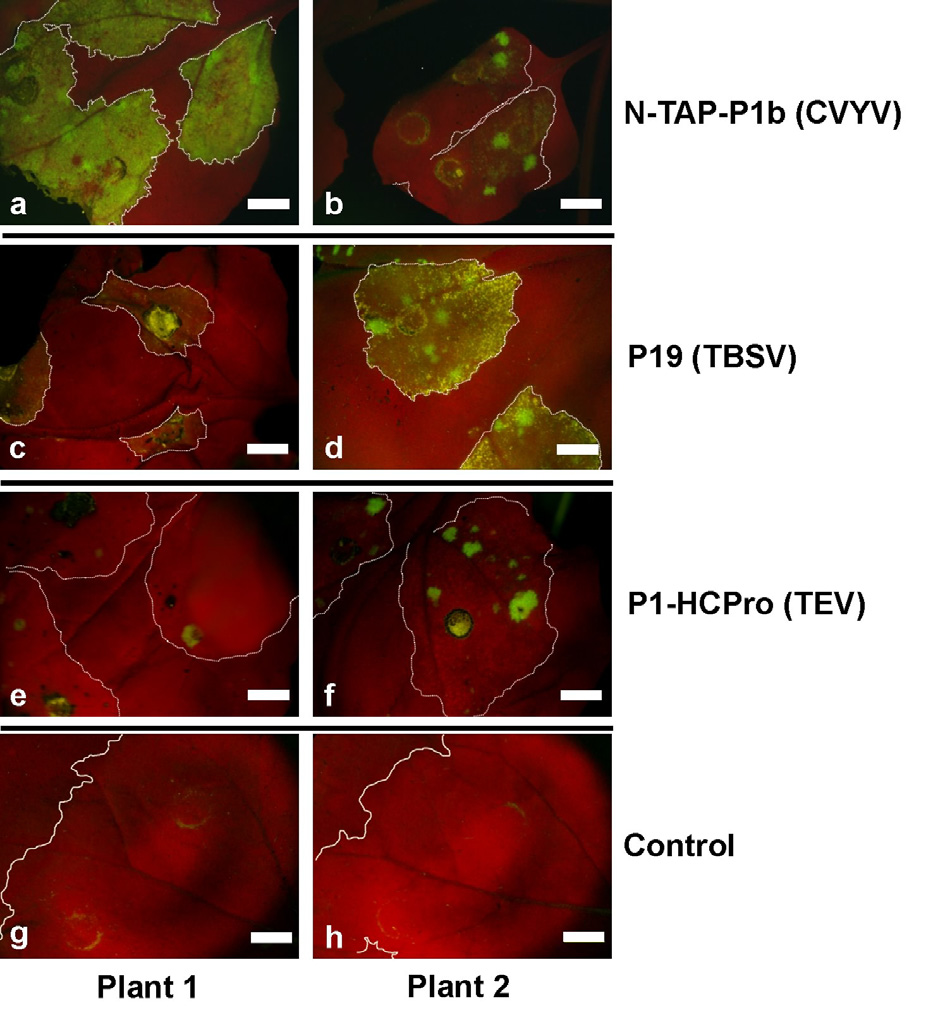
**Effect of silencing suppressors on 10.6.1 plants**. Three leaves (positions 3-5 from the bottom) of 10.6.1 plants were infiltrated with *Agrobacterium *strains expressing different RNA silencing suppressors or with a control *Agrobacterium *containing an empty vector. Two second agroinfiltrated leaves are shown under UV light at 11 days post infiltration. The agroinfiltrated area is marked with a dotted white line. Scale bar: 0.5 cm.

Expression of the silencing suppressor P1b (intact or with a N-terminal TAP tag) from *Cucumber vein yellowing virus *(CVYV) gave rise in some leaves to diffused GFP fluorescence throughout the infiltrated tissue, with some dispersed more intense green spots (Fig. 4a). Only green fluorescent spots were detected in other leaves agroinfiltrated with this silencing suppressor (Fig. 4b) and in a low percentage of leaves agroinfiltrated with the silencing suppressors P19 from *Tomato bush stunt virus *(TBSV) (Fig. 4d) or P1-HCPro from *Tobacco etch virus *(TEV) (Fig. 4f). However, GFP fluorescence was not detected in most of the leaves expressing either TBSV p19 or TEV P1-HCPro (Fig. 4c and 4e). TBSV P19 tended to cause some yellowing fluorescence, likely indicative of necrosis induction (Fig. 4d). The appearance of GFP fluorescence correlated with detection of both GFP and PPV CP in western analysis (data not shown). GFP fluorescence was never detected in tissue infiltrated with *Agrobacterium *cultures carrying a control empty vector (Fig. 4g-h), indicating that the amplicon induction is the result of the expression of the silencing suppressor.

The systemic expression of the amplicon did not appear to be enhanced in the plants agroinfiltrated with the silencing suppressors with respect to plants agroinfiltrated with the empty vector or not agroinfiltrated, suggesting that local RNA silencing suppression is not enough to relief PPV from the defense mechanisms that prevent its spread in the 10.6 amplicon plants.

### Susceptibility of the 10.6 PPV-NK-GFP amplicon plants to exogenous viral infection

Since the 10.6 plants appear to have defense mechanisms that limit the expression of the transgene amplicon, we wondered whether these antiviral activities were able to interfere with exogenous infections and whether these infections could affect the expression of the endogenous PPV amplicon.

To answer these questions, 10.6.1 plants that did not display signs of PPV-NK-GFP infection and wild type *N. benthamiana *plants were manually inoculated with either wild type PPV, a second potyvirus, *Tobacco vein mottling virus *(TVMV), or a heterologous virus, the cucumovirus CMV, or mock-inoculated.

**Figure 5:**
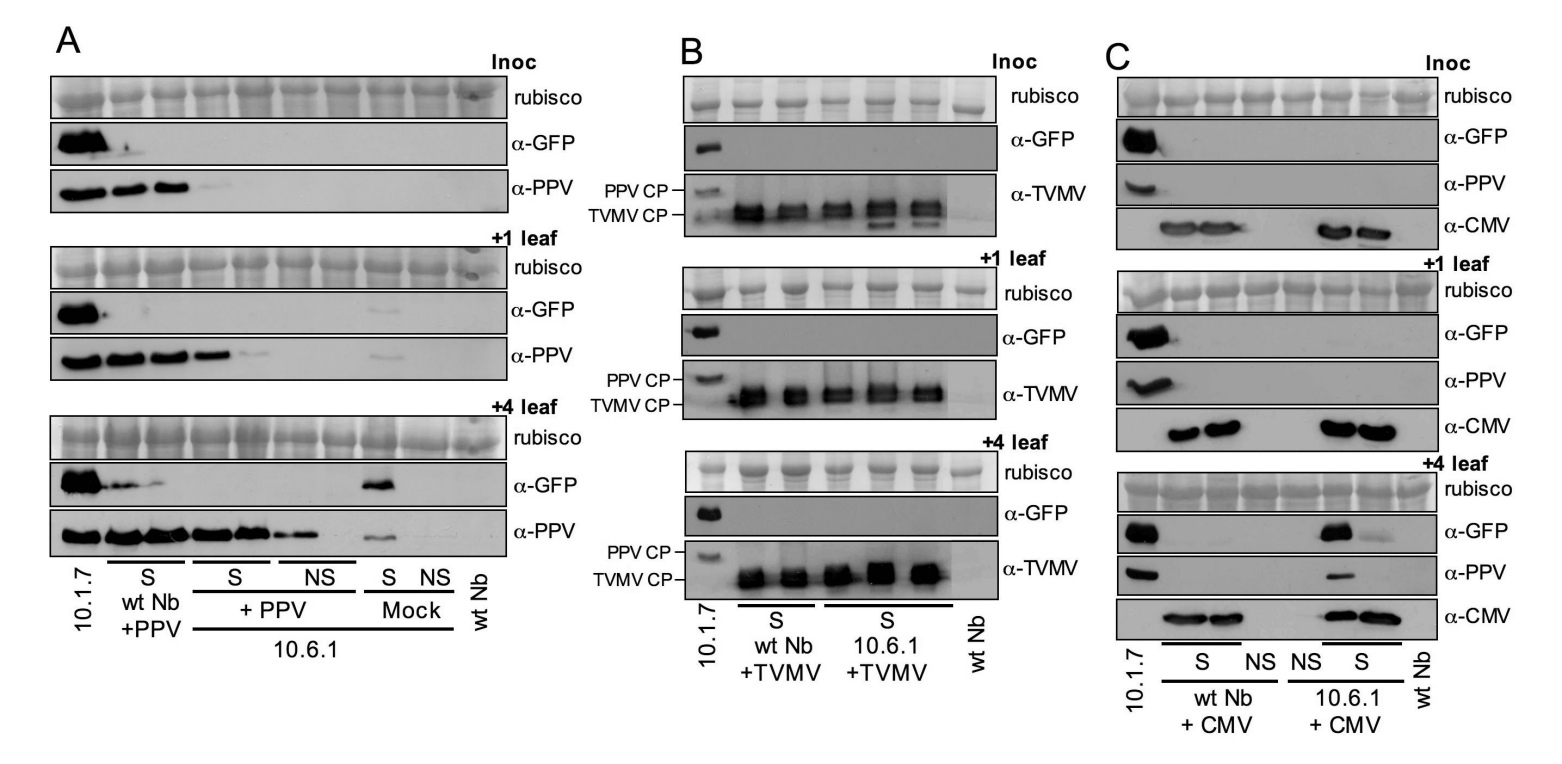
**Virus and GFP accumulation in wild type and 10.6.1 transgenic *N. benthamiana *plants inoculated with different viruses**. Extracts of inoculated leaves (inoc.), the leaf immediately above the inoculated ones (+ 1 leaf), and the fourth leaf above the inoculated ones ( + 4 leaf) from 10.6.1 transgenic or wild type *N. benthamiana *plants inoculated with PPV (panel **A**), TVMV (panel **B**) or CMV (panel **C**) displaying (S) or not displaying (NS) symptoms (either single plants or pools from two or three plants), collected at 18 days post inoculation, were subjected to western analysis with the indicated antibodies (TVMV antibody also reacts with PPV CP). One mock inoculated 10.6.1 plant that developed an spontaneous infection (S) and a pool of three of these plants that did not show symptoms of amplicon-derived infection (NS) were also analyzed (panel **A**). Samples from a 10.1.7 transgenic plant, expressing the PPV-NK-GFP amplicon from germination, and from a healthy *N. benthamiana *plant (wt Nb) were used as positive and negative controls, respectively. The blots stained with Ponceau red showing the rubisco protein are presented as loading controls.

All the plants inoculated with TVMV developed a systemic infection (Table 1). The 10.6.1 transgenic plants did not show enhanced resistance to CMV either, although some wild type and 10.6.1 plants were not infected, probably as a consequence of a low infectivity of the CMV inoculum used (Table 1). The lack of resistance of 10.6.1 plants against these viruses was confirmed by western analysis (Fig. 5). In contrast, whereas all the wild type plants were susceptible to wild type PPV infection, only 5 out of 8 10.6.1 plants inoculated with this virus displayed disease symptoms (Table 1). The partial resistance of 10.6 plants to PPV was further supported by western analysis showing much lower accumulation of PPV CP in the inoculated leaves and in the leaves immediately above them of the 10.6.1 infected plants with respect to that of the wild type ones (Fig. 5). Interestingly, this resistance appeared to be overcome with the infection progress, since the levels of PPV accumulation were similar in the fourth leaf above the inoculated ones of 10.6.1 and wild type infected plants (Fig. 5).

**Table 1:** Infectivity of different viruses in 10.6.1 PPV-NK-GFP amplicon plants.

	Inoculated plants^a^	Plants displaying disease symptoms	Plants displaying amplicon-derived infection	Recovered plants
10.6.1 + wt PPV	8	5^b^	0	5
10.6.1 + TVMV	8	8	0	0
10.6.1 + CMV	8	6	6	0
10.6.1 Mock	8	1	1	1
Wt + wt PPV	4	4	-	0
Wt + TVMV	4	4	-	0
Wt + CMV	4	2	-	0

^a ^10.6.1 transgenic (10.6.1) or wild type (wt) *N. benthamiana *plants were manually inoculated with the indicated viruses. ^b^Some PPV CP was detected by western analysis in upper non-inoculated leaves of two asymptomatic plants (Fig. 5A).

In this experiment, only one mock inoculated 10.6.1 plant developed infection from the endogenous PPV-NK-GFP amplicon. The fact that none of the 10.6.1 plants inoculated with wild type PPV showed GFP fluorescence (not shown) or GFP accumulation (Fig. 5A), indicated that systemic spread of exogenously applied PPV is not able to suppress the repression of the endogenous amplicon.

No GFP fluorescence or GFP accumulation in western blot were observed in TVMV-infected plants, showing that TVMV, like the exogenous wild type PPV, was not able to derepress the PPV amplicon of the 10.6 line (Fig. 5B). In contrast, all 10.6.1 plants infected with CMV showed GFP expression on some infected leaves. GFP appeared as small spots apparently located at random leaves, approximately seven days after these leaves developed CMV symptoms (not shown), and never displayed the typical GFP expression pattern of the amplicon-derived systemic infection of these plants (Fig. 2B). Amplicon expression in CMV-infected plants was confirmed by GFP and PPV CP detection by western analysis (Fig. 5C).

**Figure 6:**
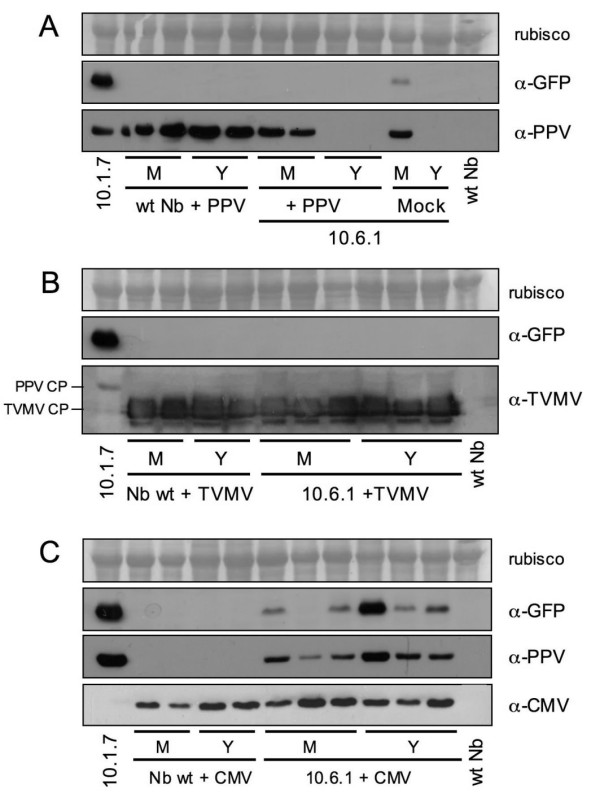
**Recovery from virus infection in 10.6.1 transgenic plants**. Extracts of mature (M) or young (Y) leaves from 10.6.1 transgenic or wild type *N. benthamiana *plants inoculated with PPV (panel **A**), TVMV (panel **B**) or CMV (panel **C**) collected at 32 days post inoculation were subjected to western analysis with the indicated antibodies (TVMV antibody also reacts with PPV CP). One mock inoculated 10.6.1 plant that developed an spontaneous infection was also analyzed (panel **A**). Samples from a 10.1.7 transgenic plant, expressing the PPV-NK-GFP amplicon from germination, and from a healthy *N. benthamiana *plant (wt Nb) were used as positive and negative controls, respectively. The blots stained with Ponceau red showing the Rubisco protein are presented as loading controls.

A typical feature of PPV infection derived from amplicon expression in 10.6 plants is the recovery at late times of the infection (Fig. 2B). Western analysis demonstrated that 10.6.1 plants could recover from the infection produced by an exogenous PPV in the same way as they recovered from an endogenous amplicon infection (Fig. 6A): CP was not detected in young leaves that were not displaying symptoms or GFP expression any more. In contrast, neither wild type *N. benthamiana *recovered from PPV infection (Fig. 6A), nor the transgenic 10.6.1 plants recovered from TVMV or CMV infection (Fig. 6B and 6C). In addition, the sporadic CMV-induced derepression of the PPV-NK-GFP amplicon still took place in young CMV-infected leaves at late times at which 10.6.1 plants infected with endogenous or exogenous PPV were already recovered (Fig. 6A and 6C).

### Virus movement from and towards PPV-NK-GFP 10.6 transgenic tissue

To gain insight into the infection step at which systemic expression of the 10.6 amplicon is prevented, we made reciprocal grafts between 10.6.1 and wild type *N. benthamiana *plants.

Type I grafts consisted on a 10.6.1 rootstock and a wild type scion (abbreviated Rx/Swt, where x is the 10.6.1 plant number). Type II grafts consisted on a wild type rootstock and a 10.6.1 scion derived from the same plants used for the reciprocal type I graft (abbreviated Rwt/Sx). None of the 10.6.1 plants expressed GFP at the time of grafting.

**Figure 7:**
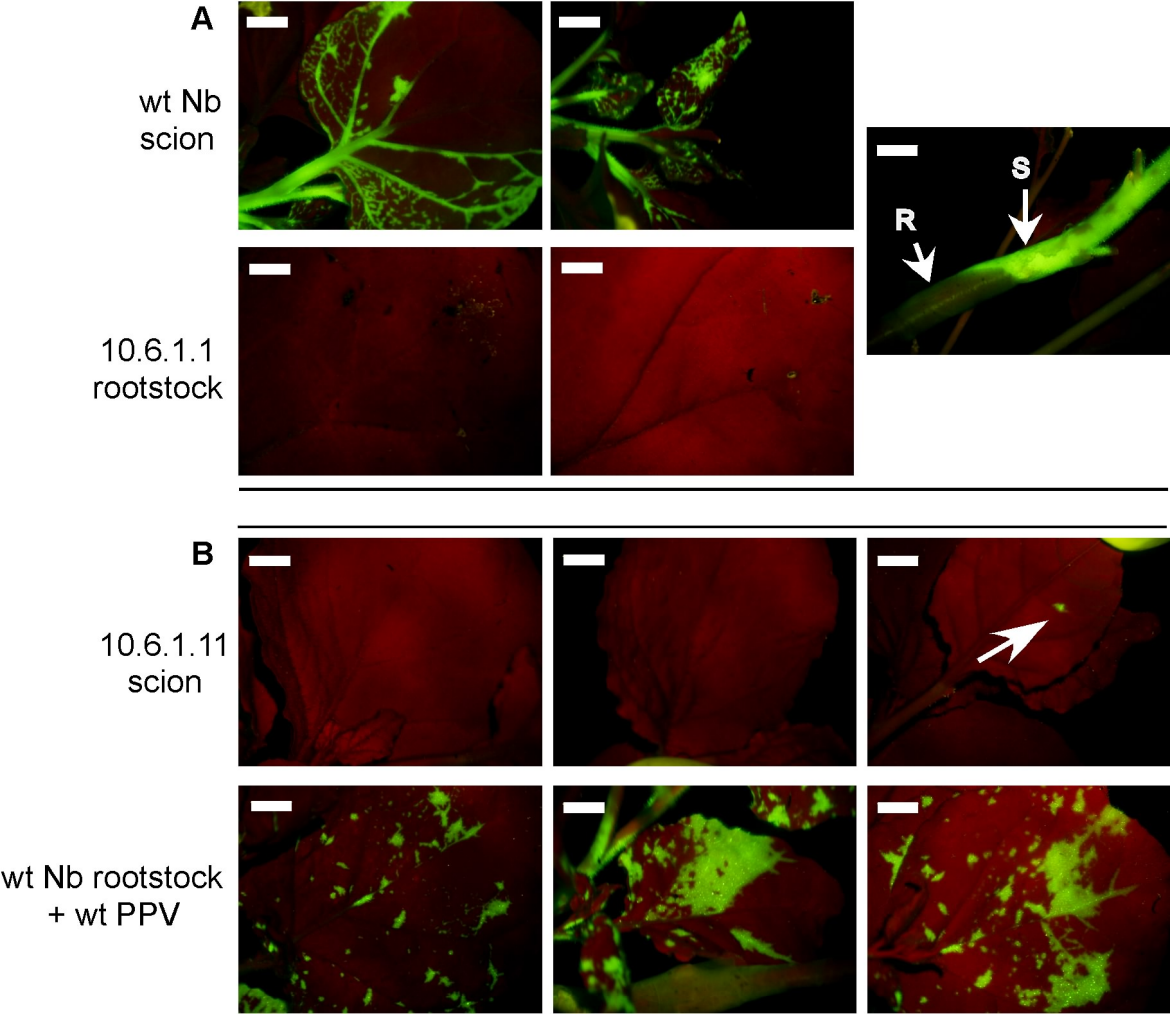
**Movement of 10.6 amplicon-derived virus through graft junctions**. Reciprocal grafts were made using wild type *N. benthamiana *and 10.6.1 transgenic plants. Rootstock and scion leaves of grafted plants consisting of a wild type scion on a 10.6.1 rootstock (**A**) and of 10.6.1 scion on a wild type rootstock that was inoculated with wild type PPV after grafting (**B**) are shown under UV light; pictures were taken at 22 (**A**) and 35 (**B**) days after grafting. The only small GFP spot detected in the 10.6.1.11 scion is indicated by an arrow. A picture of the graft junction of the R10.6.1.1/Swt plant is also shown. Note that the GFP is only visible on the wild type part of the stem and not in the transgenic. Scale bar: 0.5 cm.

**Figure 8:**
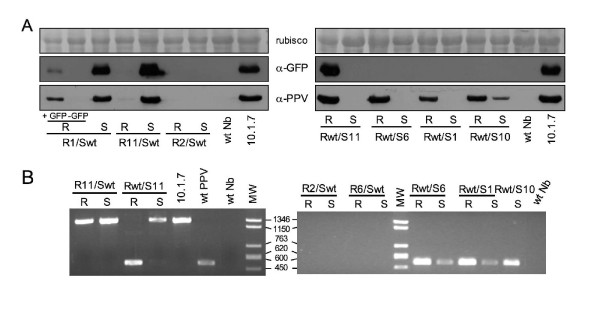
**Virus and GFP accumulation in 10.6 grafted plants**. Extracts from leaves of the scion (S) and rootstock (R) sections of grafted plants consisting of a wild type scion on a 10.6.1 rootstock or of a 10.6.1 scion on a wild type rootstock that was inoculated with wild type PPV after grafting, were subjected to western analysis with specific antibodies (**A**), as well as to IC-PCR yielding cDNA fragments of 511 nt and 1255 nt for wild type PPV and PPV-NK-GFP, respectively (**B**). In the case of the plant R1/Swt, extracts from leaves of the rootstock displaying (+ GFP) or not displaying (-GFP) GFP fluorescence were analyzed. Leaves were collected at 39 days after grafting. Samples from a 10.1.7 transgenic plant, expressing the PPV-NK-GFP amplicon from germination, and from wild type *N. benthamiana *plants healthy (wt Nb) or infected with wild type PPV (wt PPV) were used as controls. Blots stained with Ponceau red showing the Rubisco protein are presented as loading controls in western blot analyses.

Amplicon expression was only observed in two out of eight type I grafted plants (R1/Swt and R11/Swt). Surprisingly, in these grafted plants, disease symptoms and GFP fluorescence were detected in the wild type scions when still there were no signs of amplicon expression in the transgenic rootstocks (Fig. 7A and data not shown). One of these rootstocks (R1/Swt) displayed GFP and disease symptoms in newly emerging lateral branch leaves, at later times. GFP could not be detected by western analysis in the rootstock tissue that did not display green fluorescence. In the case of R11/Swt, viral RNA was amplified by IC-PCR and a faint CP band was detected in a western analysis from the asymptomatic rootstock (Fig. 8). Infection progressed in the wild type scions of the R1/Swt and R11/Swt plants in a manner similar to wild type intact plants, and did not recover from the amplicon-derived infection (data not shown). Amplicon expression was not observed in any of the other type I grafted plants, and viral products could not be detected by western analysis or IC-PCR (Fig. 8 and data not shown).

The wild type rootstocks of eight type II grafted plants were inoculated with a full-length infectious cDNA of wild type PPV at 4 days after grafting (dpg). Four plants (Rwt/S1, Rwt/S2, Rwt/S4, and Rwt/S10) displayed a wild type PPV infection in the wild type rootstocks and two of them (Rwt/S4 and Rwt/S10) showed mild PPV symptoms on a few scion leaves, which did not spread throughout the rest of the scion (data not shown). PPV CP, but not GFP, was detected by western analysis in the rootstocks and the scions displaying symptoms (Fig. 8 and data not shown). A very faint CP band was detected in the western analysis of the asymptomatic scion of the plant Rwt/S2 (not shown). Although PPV CP was not detected by western analysis in the asymptomatic scion of the plant Rwt/S1 (Fig. 8A), a diagnostic fragment from wild type PPV RNA was amplified by IC-PCR from this scion, and the same fragment was also amplified in samples from the symptomatic rootstock of this plant and the symptomatic scion of Rwt/S10 (Fig. 8B). The four type II grafted plants that were not infected with the first PPV cDNA inoculation were reinoculated with a crude extract of PPV-infected leaves at 25 dpg, when they still did not show evidence of amplicon expression. Three of them (Rwt/S6, Rwt/S7, and Rwt/S22) resembled the Rwt/S1 plants in showing disease and CP accumulation only in the rootstock (Fig. 8A and data not shown), although a cDNA fragment from wild type PPV was amplified from both the symptomatic rootstock and the asymptomatic scion of the plant Rwt/S6 (Fig. 8B). GFP fluorescence or GFP protein accumulation was not detected in any of these three plants (Fig. 8A and data not shown).

In contrast with the rest of the type II grafted plants, the plant Rwt/S11 developed a GFP-expressing amplicon-derived infection in young leaves of the wild type rootstock, only showing one small GFP spot on one leaf of the transgenic scion (Fig. 7B). Large amounts of GFP and PPV CP were detected by western analysis in the GFP-expressing symptomatic rootstock, but not in the asymptomatic scion of this plant (Fig. 8A). A cDNA fragment from wild type PPV was amplified by IC-PCR from the rootstock of the Rwt/S11 plant, showing that the plant is coinfected by the GFP-expressing amplicon-derived virus and the exogenous wild type PPV (Fig. 8B). IC-PCR of extracts from the asymptomatic Rwt/S11 transgenic scion mainly amplified the cDNA fragment of the amplicon-derived virus, but also yielded a very low amount of the fragment of wild type PPV (Fig. 8B), suggesting that the exogenous virus might have also invaded the transgenic tissue of this plant.

## Discussion

We have transformed *N. benthamiana *with the complete cDNA sequence of a GFP-expressing recombinant PPV, giving rise to different PPV amplicon lines, which displayed great expression variability (Fig. 1). Previous reports showed that a repressed amplicon could be activated by coexpression of a strong silencing suppressor [16]. Our results show that amplicon expression can be repressed even when the amplicon carries a strong silencing suppressor such as P1-HCPro.

Although all cells of the amplicon plants carry the full-length cDNA of the viral genome, even in the plants of 10.1 line, which always displayed signals of amplicon expression, amplicon-derived virus infection was not uniform throughout the plant (Fig. 1B). It is not possible to discern whether infection in a particular place of the plant is the result of endogenous expression of the transgene or of virus spread from previously infected regions of the plant. In plants of the line 10.6, amplicon-derived infection was first observed in discrete and sporadic foci (Fig. 2B). This suggests that the process "nuclear transgene transcription- mRNA processing-mRNA export to the cytoplasm-mRNA translation-mRNA replication" is inefficient, as it has been previously suggested [27,28], but also that a resistance status of the plants prevents the progression of the infection from the rare cells in which the transgene transcript has been able to initiate virus replication. The establishment of a subsequent systemic infection, with a pattern similar to that of normal PPV infections, in the vast majority of the plants of the line 10.6 displaying the localized infection foci (Fig. 2B), suggests that virus infection in the upper leaves derives from virus movement from lower leaves and that, in these plants, resistance to local spreading is a stronger restraint on the infection progression than resistance to systemic movement. Another piece of evidence that resistance to virus systemic movement is weak in the 10.6 plants was the initiation of amplicon-derived virus infection in the wild type scion of grafted plants before it could be detected in the transgenic rootstock tissue (Fig. 7). This fact also suggests that infectious virus could accumulate at very low levels (below western analysis detection limit) in at least some 10.6 plants that do not display noticeable green fluorescence; however, we have been unable to infect indicator plants like wild type *N. benthamiana*, *N. clevelandii *or *Chenopodium foetidum *with extracts of leaves that were not displaying green fluorescence of 10.6 plants, even when upper leaves of these plants were expressing the amplicon (data not shown).

We still do not know which factor triggers amplicon expression in plants of line 10.6. This took place in only a small number of 10.6 plants, and the ability to support the amplicon activation appears not to be a heritable trait since all plants of the offspring of 10.6.1 plants that either expressed or did not express the amplicon regained the inactive status, and the percentage of plants showing induction of amplicon expression was similar in both type of progenies (data not shown). Taliansky *et al. *[17] showed that an abiotic stress, expression of a silencing suppressor, or infection with different viruses activated an amplicon of PLRV. However, the activation of the PPV amplicon of the 10.6 line was apparently spontaneous, although it cannot be excluded a triggering effect by, for instance, an unnoticed type of stress. The activation of the TMV amplicon described by Siddiqui *et al. *[29] was also spontaneous, but whereas the resistance limiting the expression of the TMV amplicon was broken at an specific developmental stage of the plant (approximately 7 to 8 weeks after germination), the expression of the PPV amplicon appears to depend less on the plant age (it was activated between 2 and 6 weeks after transplantation) than on the developmental stage of particular leaves (Fig. 2C). We do not know whether there was in these leaves an enhancement of the process that leads from transgene transcription in the nucleus to virus replication initiation in the cytoplasm, or a weakening of the resistance mechanism that prevents the establishment of a PPV infection from the viral RNA product of this cascade.

What is the nature of the resistance mechanism constraining amplicon expression in 10.6 plants? The partial resistance of these plants to exogenously inoculated wild type PPV, whereas they were fully susceptible to TVMV and CMV (Figs. 5B and 6) indicates that the resistance is virus-specific. The failure to detect virus-specific siRNAs in 10.6 plants before amplicon activation (Fig. 3), as it has been reported for repressed TMV amplicon [29], suggests that strong amplicon-specific postranscriptional RNA silencing is not established in these plants. However, the fact that agroinfiltration with silencing suppressors caused some limited PPV amplification (Fig. 4) might indicate that a low profile RNA silencing could be contributing to maintain the PPV amplicon inactive in the 10.6 plants. The moderate virus replication observed in the areas agroinfiltrated with silencing suppressors was not able to facilitate the establishment of a systemic infection. Taking into account that the 10.6 plants do not appear to pose a strong constraint to virus movement (see above), this fact implies that these plants show some level of resistance not only against endogenous transcript-derived infection or mechanically inoculated virus (Fig. 5A) but also to virus exiting the vascular tissue. In agreement with this suggestion, only a low percentage of 10.6 scions were infected from infected wild type rootstocks in grafted plants (Fig. 8). Thus, the spontaneous infection of 10.6 plants should require not only either a further input of transcript-derived infectious RNA or a resistance decay in the leaves showing the first virus foci, but also a weakening of antiviral protection in the upper leaves that were subsequently systemically infected.

Although potyvirus infection has been shown to suppress RNA silencing [30], amplicon activation was not detected in 10.6 plants infected with wild type PPV or TVMV (Fig. 5A and 5B). In contrast, CMV infection caused sporadic amplicon expression in some leaves of 10.6 plants (Fig. 5C). These results are in agreement with a previous report showing that both TVMV and CMV infection reverted virus-induced silencing of *N. benthamiana *plants transformed with a PPV-derived transgene, but susceptibility to PPV was only restored in the CMV-infected transgenic plants, which suggested that TVMV infection could be eliciting an interpotyvirus cross-protection mechanism [31]. A possible contribution of wild type PPV or TVMV infection to the suppression of amplicon silencing in the 10.6 plants could have been masked by this uncharacterized cross-protection mechanism. Alternatively, CMV could suppress a resistance mechanism limiting amplicon expression of 10.6 plants different from RNA silencing. In this regard, the 2b of CMV has been shown to interfere not only with RNA silencing but also with salicylic acid-mediated virus resistance [32]. On the other hand, the fact that a CMV mutant lacking the silencing suppressor 2b was able to facilitate virus escape from a silenced PLRV amplicon, suggests that CMV could also suppress a virus resistance mechanism by a 2b-independent pathway [17]. In any case, the patchy pattern of amplicon expression observed in the CMV-infected 10.6 plants indicates that CMV infection is not able to suppress all defensive barriers that are lifted during the apparently spontaneous induction of the PPV-NK-GFP amplicon.

Once the amplicon of a 10.6 plant is activated, the systemic infection progresses in this plant similarly as in a wild type plant (Fig. 2B). However at later times, the 10.6 infected plants, but not the 10.1 transgenic or the wild type infected plants, displayed dark-green islands, which are typical of the RNA silencing-mediated recovery [23,25,33]. Then, the recovery progressed and newly developing leaves were virus-free (Fig. 6A). The recovery phenotype of 10.6 plants was specific of PPV, since it was also observed in 10.6 plants infected exogenously with wild type PPV but not in 10.6 plants infected with TVMV or CMV (Fig. 6B and 6C). Our results do not reveal whether the repression of amplicon expression in the recovered tissue is similar to that maintaining the amplicon inactive before its spontaneous induction. The absence of large amounts of virus-specific siRNAs in the recovered tissue, like in the tissue that has not undergone amplicon activation (Fig. 3B), suggests that maintenance of amplicon repression after recovery does also not rely on a strong RNA silencing status. However, the possibility exists that weak, transient or space-localized RNA silencing could be the trigger of the recovery of 10.6 plants. This would be in agreement with another report showing that antiviral RNA silencing was restricted to the marginal region of virus-free dark green tissue in tobacco plants infected with *Tomato mosaic virus *[34].

## Conclusions

In this study, we have constructed several amplicon lines derived from the potyvirus PPV and characterized in detail one of them, the line 10.6. In spite of the production of the strong silencing suppressor HCPro, the temporal and spatial patterns of expression of the PPV amplicon are very variable among different transgenic lines and individual plants of these lines. Constraints to virus amplification and local spreading, rather than to systemic movement, appear to be the main factors restricting virus infection in the transgenic line 10.6. Virus resistance in this transgenic line is virus-specific and affects not only virus amplification from the endogenous transgene, but also the infection of exogenous virus, introduced by mechanical inoculation or by grafting. In addition, the amplicon-derived antiviral devices deployed in the 10.6 plants have a delayed effect resulting in a late recovery phenotype. Our results suggest that amplicon expression can be restrained by a combination of factors, probably including an inefficient conversion from nuclear transgene transcript to cytoplasmic replicable viral RNA, RNA silencing and other defensive responses of the plant that cannot be fully counteracted by viral suppressors.

## Methods

### Construction of p35S-PPV-NK-GFP-NOSt, *Nicotiana benthamiana *transformation and plant culture

Plasmid p35S-PPV-NK-GFP-NOSt was constructed by inserting in pBin19 [35] digested with SmaI and XbaI, the PvuII-XbaI fragment of pIC-PPV-NK-GFP [36] containing the full-length PPV cDNA sequence. *N. benthamiana *leaf discs were transformed with this construct using *A. tumefaciens *inoculation, basically according to Horsh *et al. *[37] and plants were regenerated on medium containing kanamycin (100 μg/ml). Rooted plantlets were transplanted to soil and after acclimatization in a growth chamber at 22°C and 70% humidity with a 14 h/10 h light/dark cycle, the transgenic plants were transferred to a greenhouse.

In general, seeds of the transgenic plants were germinated *in vitro *in an agar-solidified medium containing kanamycin (100 μg/ml) at 23°C with a 16 h/8 h light/dark cycle. Plantlets at the 2 leaf stage (approximately 10 days after germination) were transferred to soil and cultured in a growth chamber at 22°C, and 60% humidity, with a 14 h/10 h light/dark photoperiod.

### Expression of silencing suppressors by agroinoculation

10.6.1 transgenic plants were infiltrated with *A. tumefaciens *C58C1 strain carrying pBIN61:P19 [38], p35S-P1b, p35S-NTAP-P1b [39], p35S-P1/HC-pro [40] or the empty vector pBin19. Approximately 250 μl of acetosyringone-induced *Agrobacterium *culture (OD_600 _= 0.6) were applied by syringe to the underside of three leaves when the plants were at the eight leaves stage. As a control of the activity of the silencing suppressors, mixtures of cultures of *Agrobacterium *strains carrying p35S:GFP [41] and the plasmid coding for the silencing suppressor, were coinfiltrated in wild type *N. benthamiana*.

### Virus inoculation

Two or three leaves were dusted with carborundum and inoculated by finger-rubbing with 15 μl of either DNA of pICPPV [42] (0.7 mg/ml) or of extracts from leaves of infected wild type *N. benthamiana *plants ground with 5 mM phosphate buffer (pH 7.4) (1 g in 2 ml).

### Plant grafting

Plants at approximately 10-leaves developmental stage were grafted by the traditional cleft method. For type I grafts, 10.6.1 rootstocks were prepared by removing the shoot above two healthy basal leaves and making a vertical cut, 1.5 to 2 cm long, in the center of the stem. Scions around 4 cm long were grafted after removal of all their leaves except for the two youngest ones and the trimming of its base to a "V" wedge. Type II grafts of 10.6.1 scions in wild type rootstocks were prepared in the same way, but three leaves were left on the rootstocks to facilitate the inoculation with wild type PPV. The stock/scion junctions were secured with Parafilm and the grafted plants were covered with a plastic bag for one week to prevent dehydration.

### GFP imaging

Plant leaves were screened for GFP expression with a MZ FLII (Leica Microsystems) fluorescence stereomicroscope, using excitation and barrier filters of 480/40 nm and 510 nm, respectively. Images were collected with an OLYMPUS DP70 digital camera with DP Controller and DP manager software.

### Protein detection

Leaf tissue was ground with mortar and pestle under liquid nitrogen, and stored at -80°C. Protein extracts were prepared by thawing the powder in extraction buffer (150 mM Tris-HCl, pH 7.5, 6 M urea, 2% SDS, and 5% β-mercaptoethanol) (1 ml per g of tissue). Extracts were boiled and cell debris was removed by centrifugation. Samples were resolved on 12% SDS polyacrylamide gels and electroblotted to a nitrocellulose membrane. Specific proteins were detected using anti PPV CP or anti CMV or TVMV virions polyclonal sera as primary reagent, and peroxidase-conjugated goat anti-rabbit IgG (Jackson ImmunoResearch Laboratories) as secondary reagent, or a mixture of two anti-GFP monoclonal antibodies (Roche) as primary reagent, and peroxidase-conjugated sheep anti-mouse IgG (Sigma-Aldrich) as secondary reagent. The immunostained proteins were visualized with a LiteAbLot kit (Euroclone). Ponceau red staining was used to check the global protein content of the samples.

### RNA extraction and Northern blot analysis

Leaf tissue was ground under liquid nitrogen with mortar and pestle and stored at -80°C. Nucleic acids were extracted as it has been previously described [43]. For Northern blot analysis of siRNAs, approximately 2 μg of total RNA were resolved on a 15% denaturing polyacrylamide gel (containing 7 M urea) and transferred to a nylon Hybond-N+ membrane by capillary blotting [44]. Ethidium bromide staining of the gel and methylene blue staining of the membrane were used to verify equal loading and transference. After UV cross-linking and prehybridization in UltraHyb buffer (Applied Biosystems/Ambion), blots were hybridized in the same solution with a ^32^P-labeled antisense RNA probe corresponding to the PPV P1 or NIb coding sequence, previously hydrolyzed with carbonate buffer to an average length of approximately 50 nt. Hybridization signals were detected with a Molecular Imager FX system.

### RT-PCR after immunocapture (IC-PCR)

Leaves were homogenized in 5 mM sodium phosphate buffer, pH 7.4 (2 ml per g) and incubated 2 h at 37°C and then overnight at 4°C in tubes previously coated with anti-PPV CP IgGs. After two washing steps with 16 mM sodium phosphate buffer, 0.1 M NaCl, 0.5 g/l Tween 20, pH 7.2, RT-PCR was performed in the coated tubes using the Titan RT-PCR System (Roche). Oligodeoxynucleotides 5'-TTGGGTTCTTGAACAAGC-3' and 5'-TGGCACTGTAAAAGTTCC-3' were used to amplify cDNA fragments of 1255 nt or 511 nt, of PPV-NK-GFP or wild type PPV, respectively.

## Authors' contributions

MC made all experiments with the 10.6 line shown in this article, with the scientific supervision of GD in the first steps of the work. CL constructed the plasmid and made the plant transformation and the characterization of the primary transformants, with the JMA and JLM supervision. JO and GD made the first phenotypic analyses of seedlings of the different transgenic lines. CSM collaborated in the siRNA analysis. JAG coordinated the work, participated in the discussion of all experiments and wrote the manuscript. All authors read and approved the final manuscript.
